# Statement of Retraction: MicroRNA miR-331-3p suppresses osteosarcoma progression via the Bcl-2/Bax and Wnt/β-Catenin signaling pathways and the epithelial-mesenchymal transition by targeting N-acetylglucosaminyltransferase I (MGAT1)

**DOI:** 10.1080/21655979.2026.2667630

**Published:** 2026-05-21

We, the Editors and Publisher of the journal *Bioengineered*, have retracted the following article from publication.

Bi, W., Yang, M., Xing, P., & Huang, T. (2022). MicroRNA miR-331-3p suppresses osteosarcoma progression via the Bcl-2/Bax and Wnt/β-Catenin signaling pathways and the epithelial-mesenchymal transition by targeting N-acetylglucosaminyltransferase I (MGAT1). *Bioengineered*, 13(6), 14159–14174. https://doi.org/10.1080/21655979.2022.2083855 

Following publication, internal integrity audits revealed concerns around authorship changes which were made between submission and acceptance of the article, and around the integrity of the data and reported results in the article.

When approached for an explanation, the authors provided their original data and answered our queries, but these do not sufficiently address the concerns.  

As determining authorship and contributions and verifying the validity of published work are core to the integrity of the scholarly record, we are therefore retracting the article. The authors have been informed of this decision. The authors do not agree with the retraction.

We have been informed in our decision-making by our policy on publishing ethics and integrity and COPE guidelines.  

The retracted article will remain online to maintain the scholarly record and will be digitally watermarked on each page as ‘Retracted’.  

